# Supplementary material for: Mechanical control of nuclear import by Importin-7 is regulated by its dominant cargo YAP
Source: Nat Commun. 2022 Mar 4;13:1174. doi: 10.1038/s41467-022-28693-y (PMC8897400; doi:10.1038/s41467-022-28693-y)
Supplement: Supplementary file 1 — Supplementary Information [file 41467_2022_28693_MOESM1_ESM.pdf]

Mechanical control of nuclear import by Importin-7 is regulated by its dominant cargo YAP

María García-García et al.,

Supplementary Information

	Nuclear			Cytoplasmic			
Protein	High confl.	Low confl.	T-test	High confl.	Low confl.	T-test	Z
IPO7	0.331373372	1.574510066	0.056038192	2.683972492	1.337043516	0.03269	2.59006567
IMPA1	-0.918492994	-0.540365083	0.202341527	-0.21344042	-1.445910584	0.32727302	1.61059808
XPOT	0.989328631	1.21911928	0.325903032	1.119707206	0.421651782	0.17108296	0.92784607
XPO1	-0.04208696	-0.112988003	0.466556194	-0.55572721	-1.054346354	0.20760106	0.4277181
IPO9	0.140316343	-0.288814539	0.304445944	1.119707206	0.421651782	0.31600011	0.26892454
KPNB1	0.216414599	0.10775898	0.432931718	0.194175522	-0.098596863	0.11588934	0.18411677
IPO5	-0.710923177	-0.427314081	0.314461246	0.179491734	0.357094298	0.26904509	0.10600653
KPNA3	0.793704552	0.788142828	0.497785535	0.077994941	0.120680246	0.36000307	-0.048247
TNPO3	0.671524121	0.618177925	0.486998795	-0.648715004	-0.16170581	0.47759513	-0.5403554
TNPO2	-0.650215548	-0.754039437	0.317388052	-0.979564592	-0.423040906	0.2777185	-0.6603476
KPNA2	-0.239454893	-1.010908872	0.345213047	0.428507131	0.832731969	0.21444015	-1.1756788
TNPO1	-0.508664981	-1.052817343	0.159978606	-0.532957998	0.193916138	0.0439943	-1.2710265

Supplementary Table 1. List of the importins and exportins identified in the MS analysis.

List of the importins and exportins identified in the MS analysis (Fig. 1b), sorted according to the Z-score. From left to right, the columns show the proteins ID (Protein), the average nuclear amount in high confluence (high confl.) or low confluence (low confl.), the t-test p-value obtained from the comparison of high confl. nuclear and low confl. nuclear (T-test, in column 4), the average cytosolic amount in high confluence (high confl.) and low confluence (low confl.), the t-test p-value obtained from comparing high confl. cytosol and low confl. cytosol (T-test, column 7) and the Z-score value (Z; explained in figure legend 1b). Statistical analysis with a two-tailed unpaired t test. Raw data is provided in Supplementary Data 1.

TMT_label	Sample
127C	Cyt1
128N	Cyt2
128C	Nuc1
129N	Nuc2
129C	Cyt3
130N	Cyt4
130C	Nuc3
131	Nuc4
126	Internal standard
127N	Internal standard

Supplementary Table 2. TMT-isobaric labelling.

Scheme of the TMT-isobaric labelling of the samples and the internal standard corresponding to experiment shown in figure 1a, b.

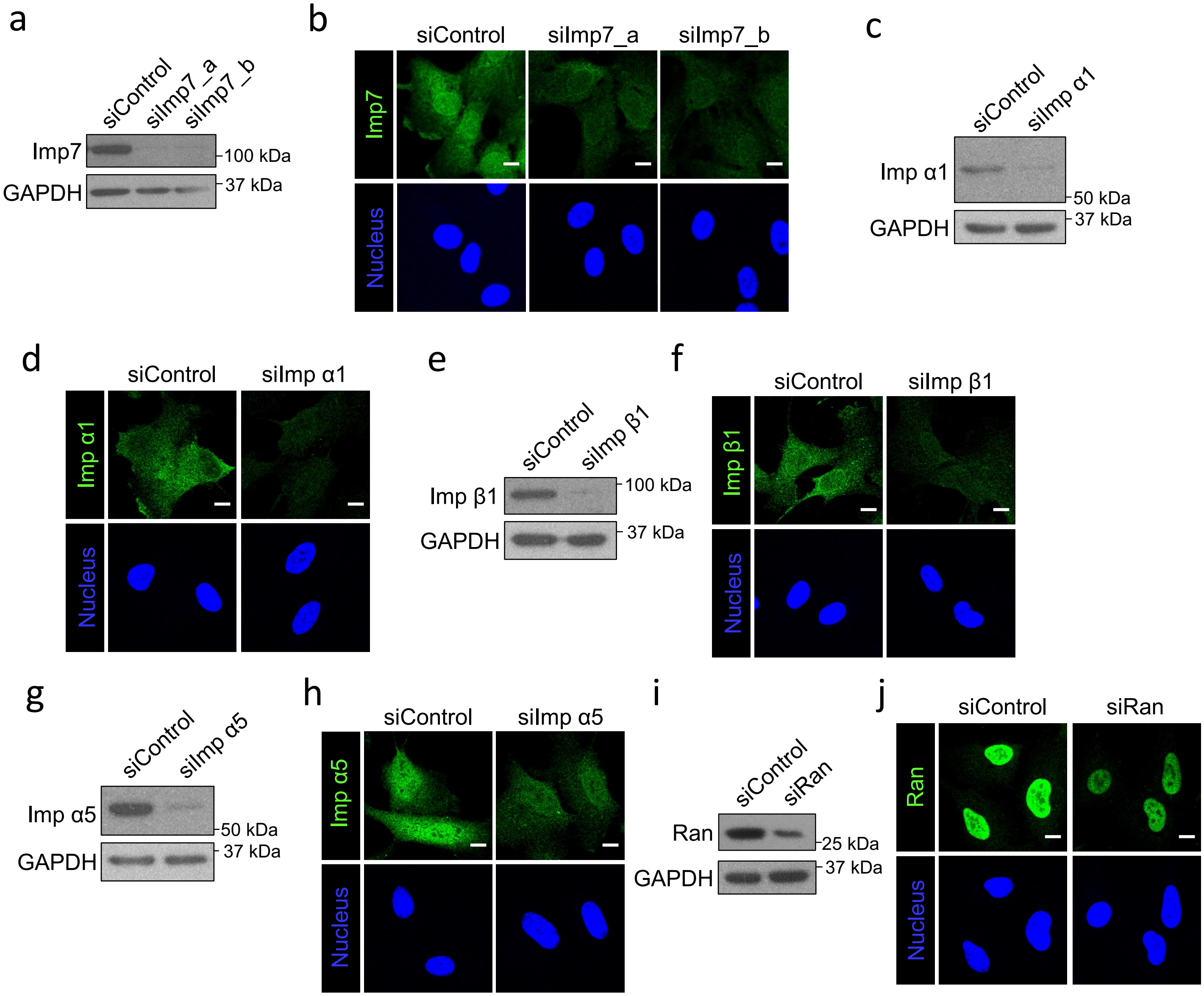

Supplementary Fig. 1. Antibody validation using specific siRNAs against the protein of interest.

a-j) In the a, c, e, g, i panels the immunoblot of the indicated proteins upon silencing with the indicated siRNAs is shown. GAPDH was used as loading control. In the adjacent panels to the immunoblots (b, d, f, h and j) the staining of the protein of interest is shown in control and cells treated with the corresponding siRNA. Representative of 3 independent experiments. Scale bar 10 µm.

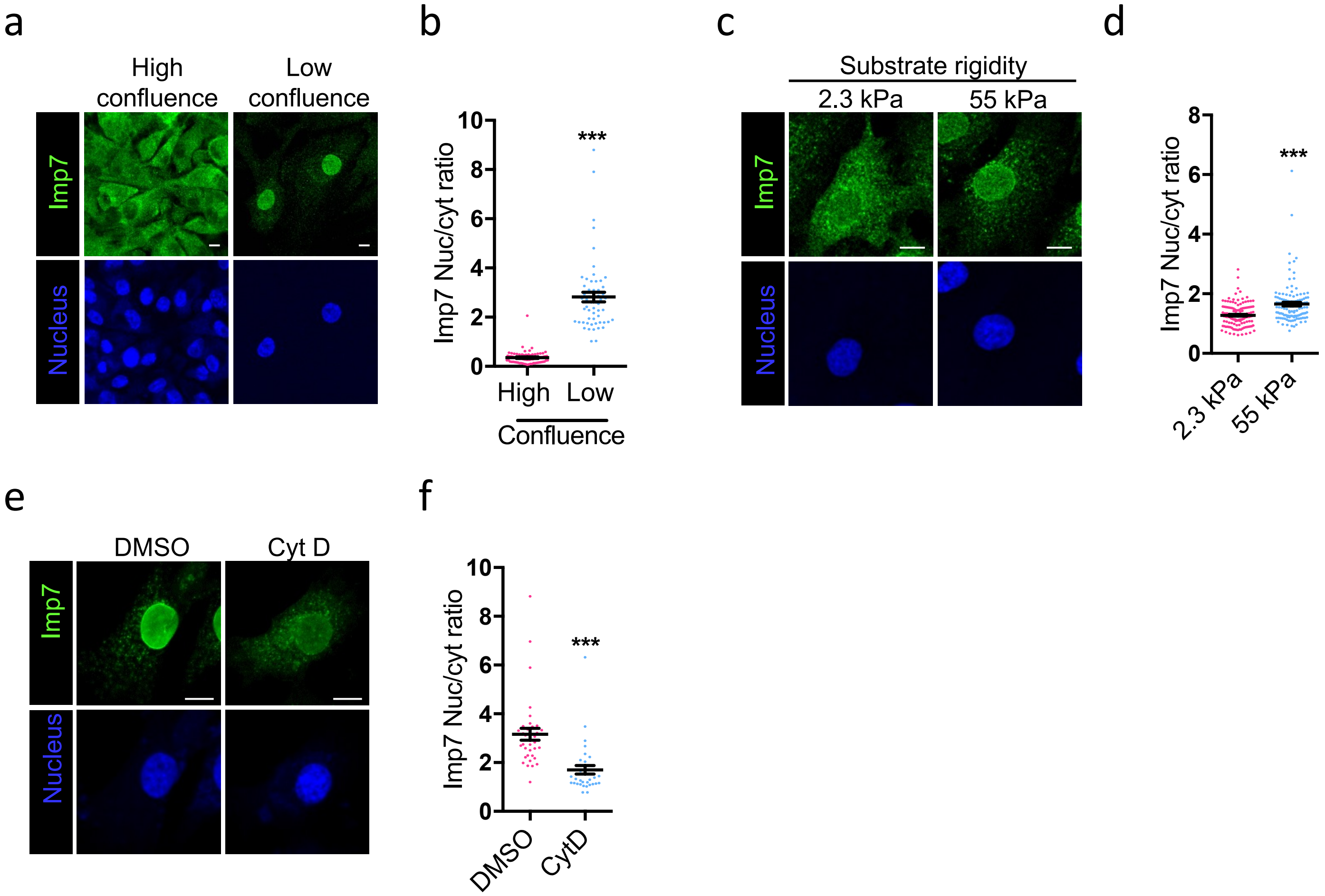

Supplementary Fig. 2. Cell density, substrate rigidity and the actin cytoskeleton regulate Imp7 nuclear accumulation in mesenchymal stem cells.

a, b) Immunofluorescence of endogenous Imp7 in MSCs plated at high (218,750 cells/cm2) and low (10,417 cells/cm2) confluence. Quantification is shown in the right graph (b). N = 76 cells in the high confluence condition and N = 57 cells in the low confluence condition from 3 independent experiments. P-value = 2.401e-18.

c, d) Immunofluorescence of endogenous Imp7 in MSCs plated on top of soft substrates (2.3 kPa) or stiff substrates (55 kPa). Quantification is shown in the right graph (d). N = 137 cells in soft condition and 123 cells in stiff matrix from 3 independent experiments. P-value = 1.958e-07.

e, f) Immunofluorescence of endogenous Imp7 and nuclei in MSCs upon cytochalasin D (Cyt D) treatment. Quantification is shown in graph (f). N = 36 and 34 cells from a representative experiment of three independent experiments. Statistical analysis with a two-tailed unpaired t test. Data represent mean ± s.e.m. Scale bar 10 µm. P-value = 6.814e-06. P-values below or equal to 0.05, 0.01 or 0.005 were considered statistically significant and were labeled with 1, 2 or 3 asterisks respectively. Raw data available in the Source Data file.

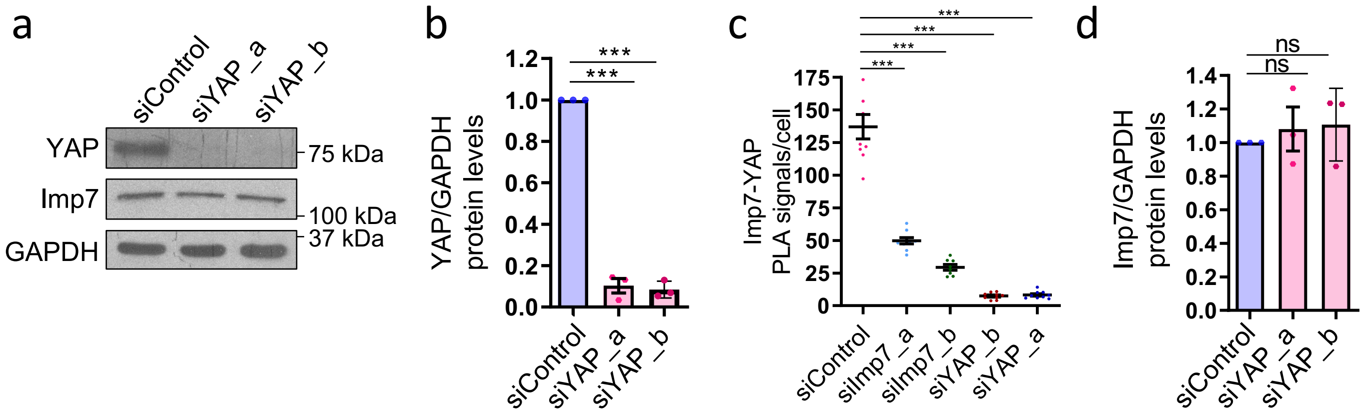

Supplementary Fig. 3. YAP silencing efficiency and specificity of the Imp7-YAP association measured by PLA.

a, b) Immunoblot and quantification showing specific depletion of YAP with two independent siRNAs. GAPDH was used as loading control. Quantification of the levels of YAP/GAPDH is shown in graph (b). N = 3 biologically independent experiments. From left to right, p-values = 0.0015 and 0.0006.

c) *In situ* PLA detection of the interaction between endogenous YAP and Imp7 in RPE-1 cells silenced with control, two independent siRNAs for Imp7 or two independent siRNAs for YAP. Cells were stained for anti-YAP (63.7) and anti-Imp7 (rabbit) antibodies. N = 9 fields from 3 independent experiments for each sample. From left to right, p-values = 7.534e-06, 1.539e-06, 6.259e-07 and 6.165e-07.

d) Quantification of the levels of Imp7/GAPDH upon YAP silencing. N = 3 biologically independent experiments. Statistical analysis with a two-tailed unpaired t test. Data represent mean ± s.e.m. P-values below or equal to 0.05, 0.01 or 0.005 were considered statistically significant and were labeled with 1, 2 or 3 asterisks respectively. Raw data available in the Source Data file.

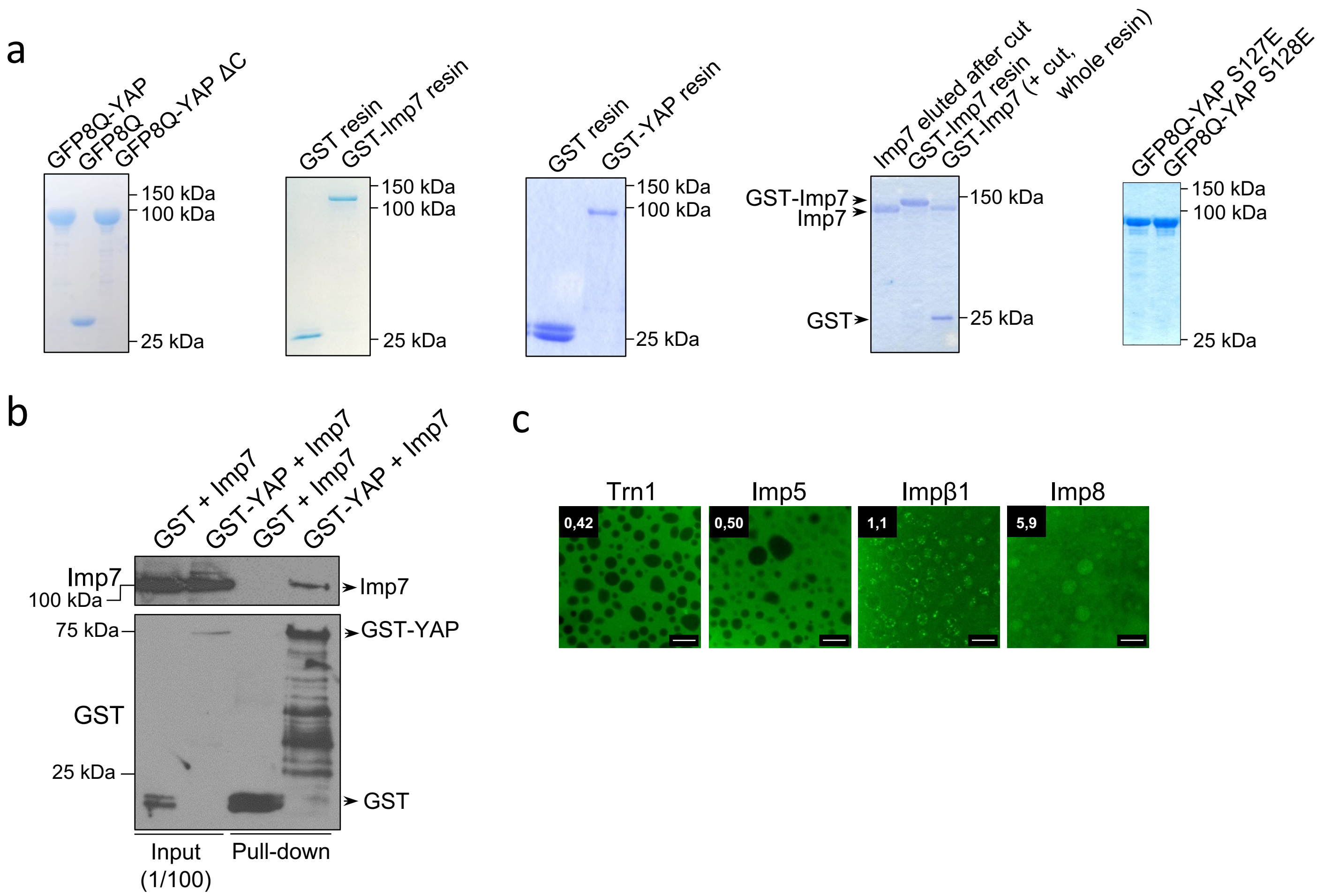

Supplementary Fig 4. Purified proteins and direct interaction of YAP and Imp7 and partitioning of YAP into FG particles.

a) Coomassie staining showing purified GFP8Q-YAP, GFP8Q-YAP ΔC, GFP8Q-YAP S127E, GFP8Q-YAP S128E, GFP8Q, GST resin, GST-Imp7 resin, GST-YAP resin, Imp7 and cut and uncut GST-Imp7 resin. Representative of 3 biologically independent experiments.

b) Direct binding of Imp7 and GST-YAP. Sepharose beads bound to GST or GST-YAP were incubated with pure Imp7 and, after extensive washing, samples were loaded in an SDS-PAGE. A fraction of the mixes (input) and the pulled-down fractions were blotted for the indicated antibodies. Representative of 3 biologically independent experiments.

c) Partitioning of GFP8Q-YAP with various NTRs into FG particles derived from scNup116 FG domain. Brightness of scans was individually adjusted. Measured partition coefficients are indicated in white inside black boxes. Scale bar 10 µm. Representative of 3 independent experiments.

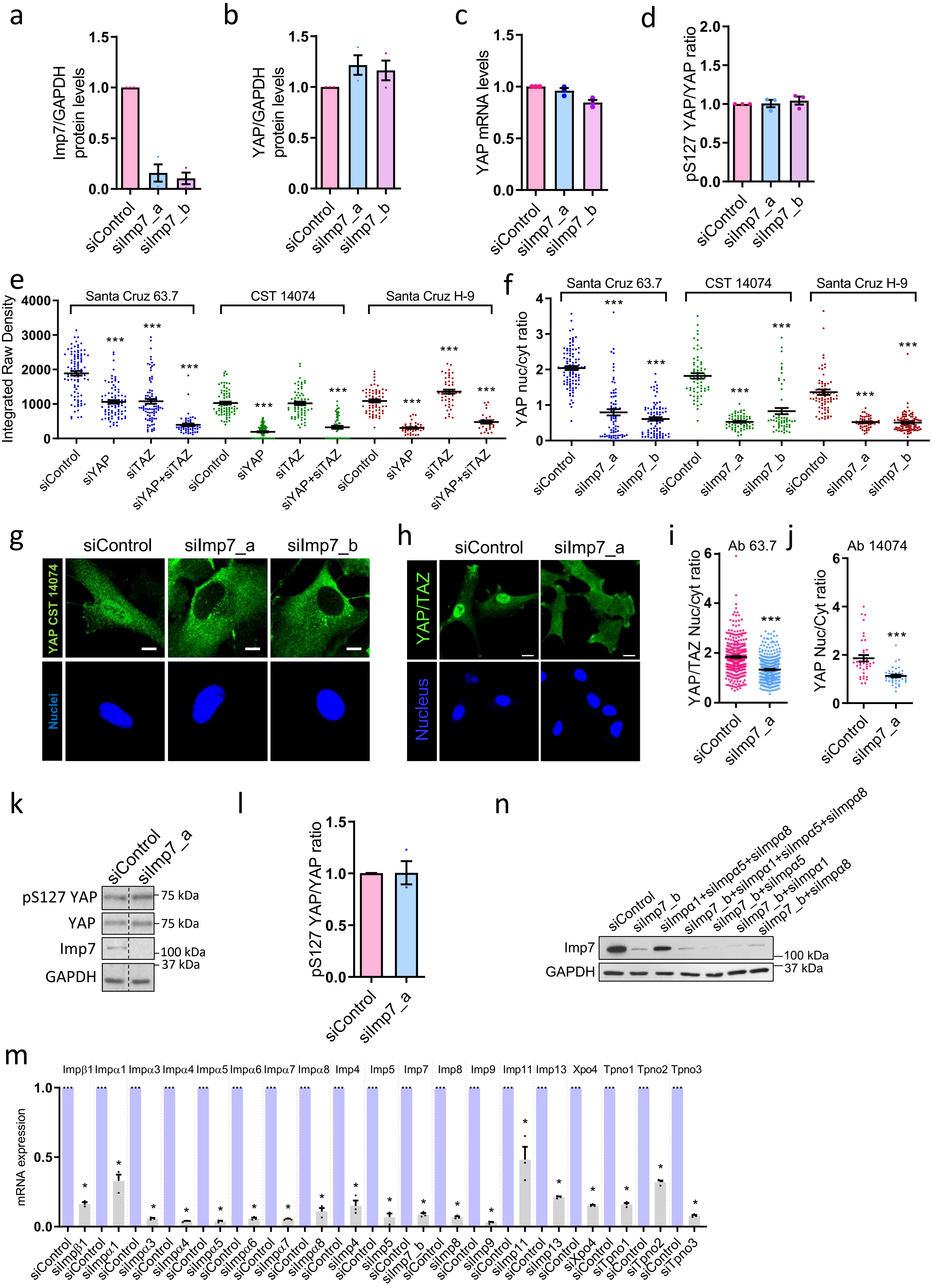
a
Imp7/GAPDH protein levels
1.5
1.0
0.5
0.0
siControl
siImp7_a
siImp7_b
b
YAP/GAPDH protein levels
c
YAP mRNA levels
d
pS127 YAP/YAP ratio
2.0
e
Integrated Raw Density
4000
3000
2000
1000
0
Santa Cruz 63.7
CST 14074
Santa Cruz H-9
***
siYAP
siTAZ
siYAP+siTAZ
f
YAP nuc/cyt ratio
g
YAP CST 14074
Nuclei
h
YAP/TAZ
Nucleus
i
Ab 63.7
YAP/TAZ Nuc/cyt ratio
j
Ab 14074
YAP Nuc/Cyt ratio
k
pS127 YAP
YAP
Imp7
GAPDH
75 kDa
100 kDa
37 kDa
l
n
siImpα1+siImpα5+siImpα8
siImp7_b+siImpα1+siImpα5+siImpα8
siImp7_b+siImpα5
siImp7_b+siImpα1
siImp7_b+siImpα8
m
mRNA expression
Impβ1 Impα1 Impα3 Impα4 Impα5 Impα6 Impα7 Impα8 Imp4 Imp5 Imp7 Imp8 Imp9 Imp11 Imp13 Xpo4 Tpno1 Tpno2 Tpno3
*
siImpβ1
siImpα1
siImpα3
siImpα4
siImpα5
siImpα6
siImpα7
siImpα8
siImp4
siImp5
siImp8
siImp9
siImp11
siImp13
siXpo4
siTpno1
siTpno2
siTpno3

Supplementary Fig 5. Imp7 regulates the nuclear accumulation of YAP in cells.

a) Quantification of the ratio between Imp7 protein levels and GAPDH levels (from Fig. 5a). The ratio was normalized to siControl. Data from 3 independent experiments.

b) Quantification of the ratio between YAP protein levels and GAPDH levels (from Fig. 5a). The ratio was normalized to siControl. Data from 3 independent experiments.

c) qRT-PCR analysis of YAP expression in RPE-1 cells silenced with control or two independent Imp7 siRNAs. Data were normalized to cells silenced with control siRNA. Data from 3 independent experiments.

d) Quantification of pS127 YAP normalized to the total YAP protein levels upon silencing of Imp7 with two independent siRNAs. The ratios were normalized to siControl. Data from 3 independent experiments.

e) Quantification of the cell signal obtained in RPE-1 cells by the staining of YAP or YAP/TAZ with the indicated antibodies and upon YAP, TAZ or YAP/TAZ silencing. Data from 3 independent experiments. From left to right, N = 94, 84, 84, 50, 73, 105, 66, 60, 63, 40, 47 and 28 cells from 3 independent experiments. From left to right, p-value = 4.69e-19, 5.07e-15, 7.27e-42, 1.50e-31, 0.887, 4.78e-22, 2.07e-27, 0.0004 and 7.86e-14.

f) Quantification of the nucleo-cytoplamic ratio obtained in RPE-1 cells by the staining of YAP or YAP/TAZ with the indicated antibodies and upon Imp7 silencing. Data from 3 independent experiments. From left to right, N = 94, 74, 82, 63, 57, 58, 73, 56 and 95 cells from 3 independent experiments. From left to right, p-value = 7.23e-23, 3.14e-44, 7.77e-26, 9.16e-14, 7.20e-17 and 4.01e-17.

g) Immunofluorescence of YAP in RPE-1 cells using the indicated antibody upon silencing of Imp7 with two independent siRNAs.

h, i) Immunofluorescence of endogenous YAP/TAZ stained with Santa Cruz 63.7 antibody (Ab 63.7) in MSCs silenced with Imp7 siRNA. Quantification is shown in graph S5i. N = 316 cells (siControl) and 359 (siImp7), from 3 independent experiments. P-value = 4.62e-24.

j) Quantification of the immunofluorescence of endogenous YAP stained with Cell Signaling Technology 14074 antibody (Ab 14074) in MSCs silenced with Imp7 or control siRNA. N = 38 cells (siControl) and 38 (siImp7), from 3 independent experiments. P-value = 5.83e-06.

k) Immunoblot showing specific suppression of Imp7 in MSCs. GAPDH was used as loading control. Samples were run in two separate gels and blotted for the indicated antibodies. Note that YAP and pS127 YAP blots were performed in different gels simultaneously. Irrelevant lanes in the blot were removed and denoted by the dotted line. Representative of 3 biologically independent experiments.

l) Quantification of the ratio between pS127 YAP protein levels and YAP levels from panel k. The ratio was normalized to siControl. N = 3 biologically independent experiments.

m) qRT-PCR of the indicated mRNAs after the corresponding silencing. Data represent mean ± sem. Data from 3 independent experiments. From left to right, p-value = 0.019, 0.044, 0.048, 0.049, 0.020, 0.043, 0.016, 0.047, 0.020, 0.028, 0.041, 0.035, 0.025, 0.030, 0.047, 0.025, 0.017, 0.034 and 0.041.

n) Immunoblot showing specific suppression of Imp7 in RPE-1 cells upon different combinations of the indicated siRNAs. GAPDH was used as loading control. Statistical analysis with a two-tailed unpaired t test. Data represent mean ± sem. Representative of 3 biologically independent experiments. Scale bar 10 µm. P-values below or equal to 0.05, 0.01 or 0.005 were considered statistically significant and were labeled with 1, 2 or 3 asterisks respectively. Raw data available in the Source Data file.

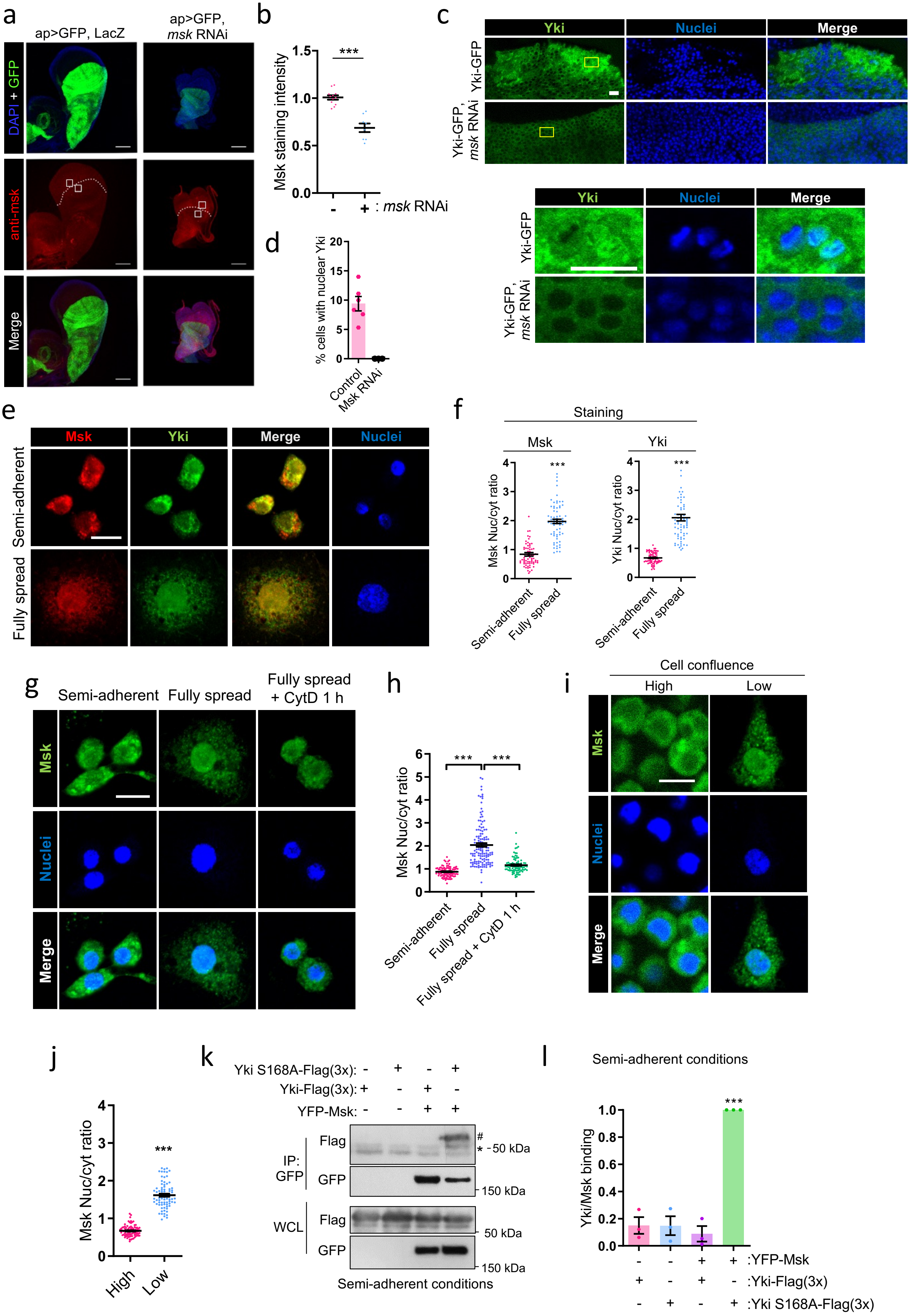

a
ap>GFP, LacZ
ap>GFP, msk RNAi
DAPI + GFP
anti-msk
Merge
b
Msk staining intensity
***
- + : msk RNAi
c
Yki
Nuclei
Merge
Yki-GFP
Yki-GFP, msk RNAi
d
% cells with nuclear Yki
Control
Msk RNAi
e
Msk
Yki
Merge
Nuclei
Semi-adherent
Fully spread
f
Staining
Msk
Yki
Msk Nuc/cyt ratio
Yki Nuc/cyt ratio
***
Semi-adherent
Fully spread
g
Semi-adherent
Fully spread
Fully spread + CytD 1 h
Msk
Nuclei
Merge
h
Msk Nuc/cyt ratio
***
Semi-adherent
Fully spread
Fully spread + CytD 1 h
i
Cell confluence
High
Low
Msk
Nuclei
Merge
j
Msk Nuc/cyt ratio
***
High
Low
k
Yki S168A-Flag(3x): - + - +
Yki-Flag(3x): + - + -
YFP-Msk: - - + +
IP: GFP
Flag
GFP
WCL
Flag
GFP
#
*
-50 kDa
150 kDa
50 kDa
150 kDa
Semi-adherent conditions
l
Semi-adherent conditions
Yki/Msk binding
***
- - + + :YFP-Msk
+ - + - :Yki-Flag(3x)
- + - + :Yki S168A-Flag(3x)

Supplementary Fig. 6. Silencing of Msk in *Drosophila,* localization of Yki upon Msk knockdown and Msk localization in S2 cells under conditions that regulate cell tension.

a, b) Confocal images of *Drosophila* imaginal discs overexpressing *GFP* + *LacZ* and *GFP* + *msk* siRNA under the UAS/Gal4 system in the dorsal compartment (under the control of apterusGal4). Anti-Msk staining is shown in red. The dotted line indicates the edge between compartments and the white squares the locations where the mean pixel intensity was measured. (b) Quantification of the Msk signal ratio between the region control and silenced for *msk* in the apterus compartment, in both conditions, is shown. N = 12 (-) and 8 (+) areas from 3 independent experiments. P-value = 7.37e-05. Scale bar 100 µm.

c) Confocal images of *Drosophila* imaginal discs overexpressing *Yki-GFP* and *msk* RNAi. Lower panels are a ROI of a region in the upper panels. Data representative from 3 independent experiments. Scale bar 10 µm.

d) Quantification of nuclear Yki-GFP represented as the percentage of cells with nuclear Yki-GFP in control and *msk* RNAi samples in (c). Cells with Yki-GFP nucleo-cytoplasmic ratio above 1 were considered as cells with nuclear Yki. N = 6 regions per condition from a representative experiment of three independent experiments. P-value = 0.00061.

e, f) Immunofluorescence of endogenous Msk, Yki and nuclei in S2 cells growing without substrate (semi-adherent) or in concanavalin A (ConA, 0.5 mg/ml) coated surfaces (fully spread). Quantification of each antibody signal is shown in graph (f). N = 73 (semi-adherent) or 69 (fully spread) cells per condition, from 3 independent experiments. P-value = 3.56e-22 (Msk) and 1.79e-19 (Yki). Scale bar 10 µm.

g, h) Immunofluorescence of endogenous Msk and nuclei in S2 cells plated for 2 min (semi-adherent) or 24h (fully spread) in concanavalin A (ConA, 0.5 mg/ml) coated surfaces. Cytochalasin D was added to reduce tension in the cell for 1 h. Quantification of each signal is shown in graph (h). N = 96 (semi-adherent), 135 (fully spread) and 88 (fully spread + Cyt D) cells per condition, from 3 independent experiments. P-value, from left to right, is 5.95e-30 and 0.0022. Scale bar 10 µm.

i, j) Immunofluorescence of endogenous Msk and nuclei in fully spread S2 cells plated for 24h in concanavalin A coated surfaces at low or high confluency. Quantification of each signal is shown in graph (j). N = 88 (high) and 78 (low) cells per condition, from 3 independent experiments. P-value = 2.51e-41. Scale bar 10 µm.

k, l) S2 cells overexpressing different combinations of Msk-YFP, Yki-Flag(3x), YkiS168A-Flag(3x) and Yki ΔC-Flag(3x) were grown without substrate, therefore in semi-adherent conditions. Lysates were made and Msk-YFP was immunoprecipitated with an anti-GFP antibody. The immunopurified complexes and total cell lysates were immunoblotted with anti-Flag and anti-GFP antibodies as indicated. IgG was marked with an asterisk and Yki-Flag(3x) with a #. Quantification from three independent co-immunoprecipitations is shown in (l). P-value = 0.0039. Statistical analysis was performed with a two-tailed unpaired t test. Data represent mean ± s.e.m. P-values below or equal to 0.05, 0.01 or 0.005 were considered statistically significant and were labeled with 1, 2 or 3 asterisks respectively. Raw data available in the Source Data file.

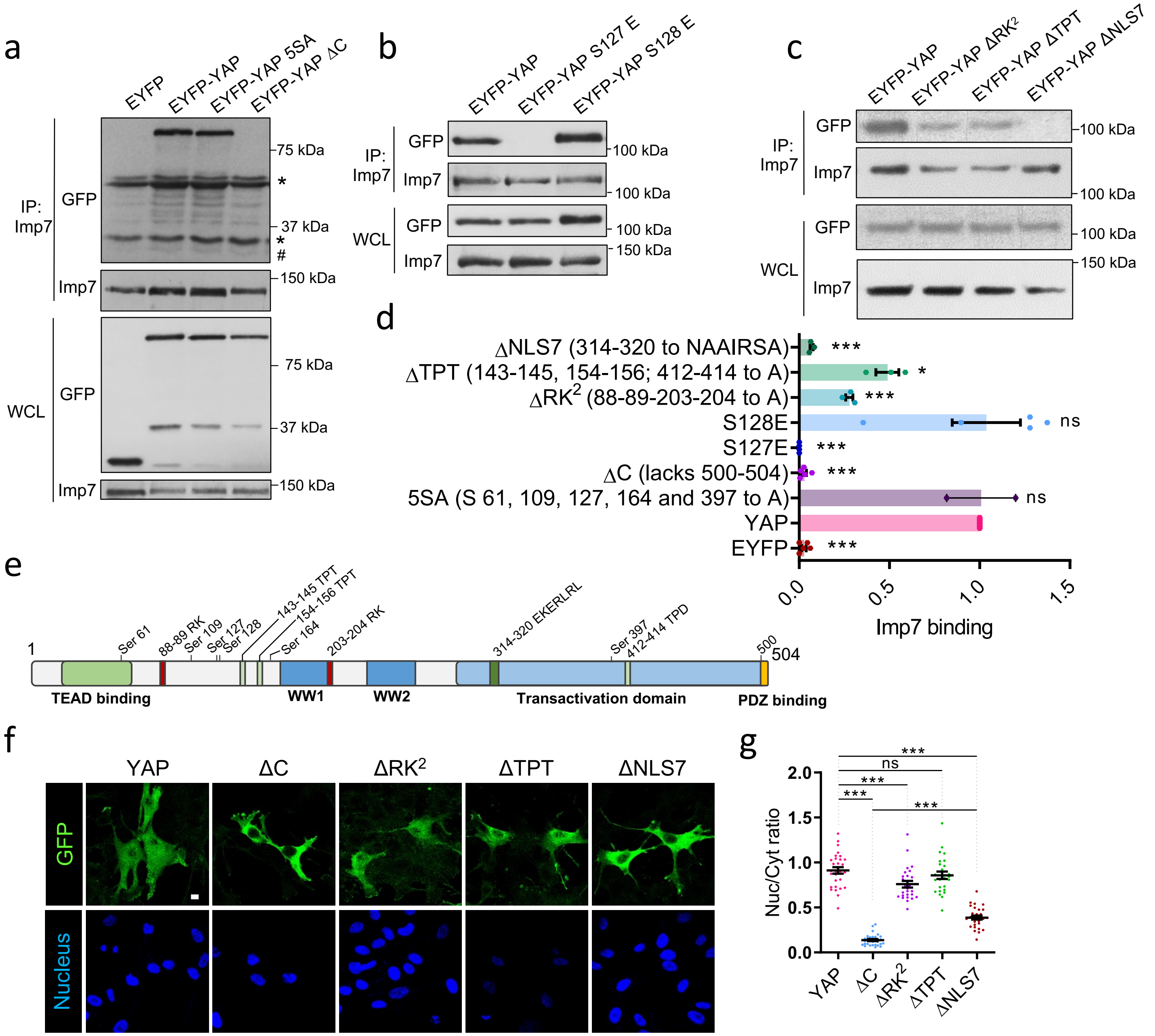

Supplementary Fig. 7. Regions in YAP required to interact with Imp7 in cells and needed for nuclear accumulation.

a, b, c) RPE-1 cells stably expressing the indicated proteins were lysed and endogenous Imp7 was immunoprecipitated. The immunopurified complexes and total cell lysates were immunoblotted with anti-GFP and anti-Imp7 antibodies as indicated. * indicates heavy and light IgG chains. # indicates the region where GFP runs, and it is not detected in the Imp7 immunoprecipitated fractions.

d) Quantification of experiments S7a, b and c. YAP and the indicated mutants correspond to EYFP-YAP versions. N = 3, 3, 3, 5, 4, 5, 2, 8, 5 immunoprecipitations per condition. Statistical analysis with a two-tailed unpaired t test of each protein against YAP. From top to bottom of the graph, p-values = 8.17e-05, 0.015, 0.00078, 0.854, 3.44e-29, 1.31e-07, 0.970 and 1.51e-07.

e) Schematic representation of the YAP protein showing known domains and relevant regions/amino acids mutated in this study.

f, g) Immunofluorescence of GFP fused to the indicated proteins and nuclei in RPE-1 cells overexpressing the indicated EYFP-YAP constructs. Quantification is shown in graph (g). From left to right in the graph, N = 29, 30, 26, 26 and 28 cells per condition, from 3 independent experiments. Statistical analysis with a two-tailed unpaired t test. Data represent mean ± s.e.m. P-values = 1.04e-20 (1st vs 2nd lanes), 0.0042 (1st vs 3rd lanes), 0.322 (1st vs 4th lanes), 1.96e-16 (1st vs 5th lanes) and 3.13e-12 (2nd vs 5th lanes). P-values below or equal to 0.05, 0.01 or 0.005 were considered statistically significant and were labeled with 1, 2 or 3 asterisks respectively. Scale bar 10 µm. Raw data available in the Source Data file.

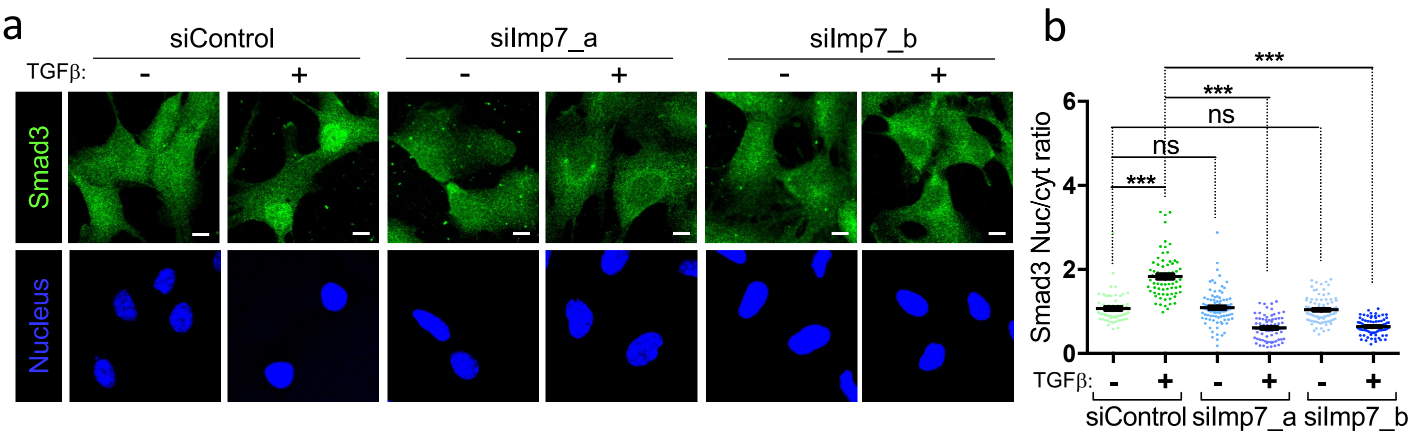

Supplementary Fig. 8. Effects of Imp7 knockdown on Smad3 nuclear accumulation.

a, b) Immunofluorescence of endogenous Smad3 in RPE-1 cells growing at low confluence and silenced for Imp7 with two independent siRNAs and treated with (+) or without (-) TGFβ. Quantification is shown in graph (b). From left to right in the graph, N = 65, 77, 95, 74, 89 and 81 cells per condition, from 3 independent experiments. Statistical analysis with a two-tailed unpaired t test. Data represent mean ± s.e.m. Scale bar 10 µm. P-values = 2.35e-19 (1st vs 2nd lanes), 0.752 (1st vs 3rd lanes), 0.517 (1st vs 5th lanes), 1.32e-36 (2nd vs 4th lanes) and 7.96e-35 (2nd vs 6th lanes). P-values below or equal to 0.05, 0.01 or 0.005 were considered statistically significant and were labeled with 1, 2 or 3 asterisks respectively. Raw data available in the Source Data file.

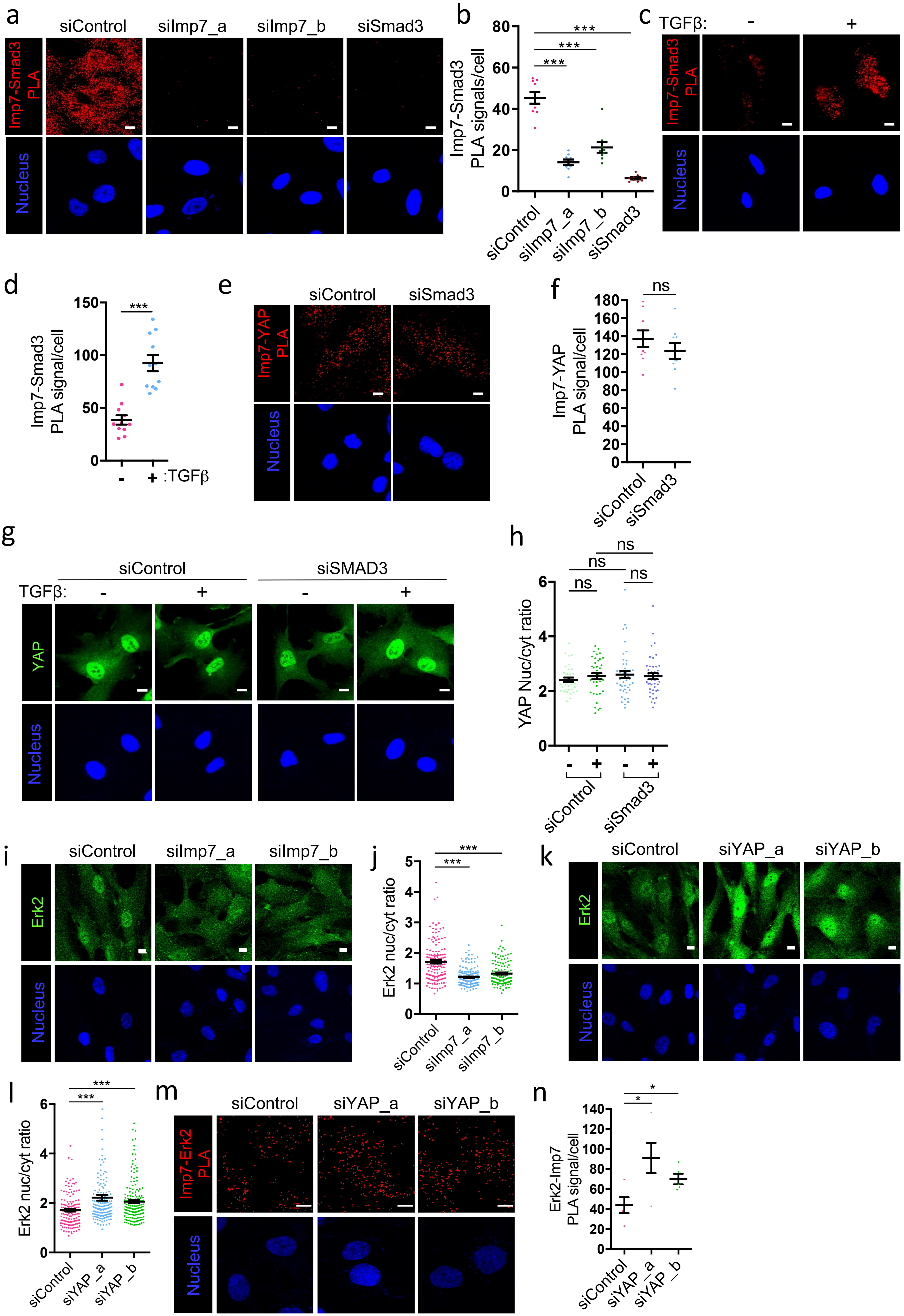
a
siControl
siImp7_a
siImp7_b
siSmad3
Imp7-Smad3 PLA
Nucleus
b
Imp7-Smad3 PLA signals/cell
80
60
40
20
0
***
***
***
siControl
siImp7_a
siImp7_b
siSmad3
c
TGFβ:
-
+
Imp7-Smad3 PLA
Nucleus
d
Imp7-Smad3 PLA signal/cell
150
100
50
0
***
-
+
:TGFβ
e
siControl
siSmad3
Imp7-YAP PLA
Nucleus
f
Imp7-YAP PLA signal/cell
180
160
140
120
100
80
60
40
20
0
ns
siControl
siSmad3
g
siControl
siSMAD3
TGFβ:
-
+
-
+
YAP
Nucleus
h
YAP Nuc/cyt ratio
6
4
2
0
ns
ns
ns
ns
-
+
-
+
siControl
siSmad3
i
siControl
siImp7_a
siImp7_b
Erk2
Nucleus
j
Erk2 nuc/cyt ratio
5
4
3
2
1
0
***
***
siControl
siImp7_a
siImp7_b
k
siControl
siYAP_a
siYAP_b
Erk2
Nucleus
l
Erk2 nuc/cyt ratio
6
4
2
0
***
***
siControl
siYAP_a
siYAP_b
m
siControl
siYAP_a
siYAP_b
Imp7-Erk2 PLA
Nucleus
n
Erk2-Imp7 PLA signal/cell
140
120
100
80
60
40
20
0
*
*
siControl
siYAP_a
siYAP_b

Supplementary Fig. 9. Analysis of different complexes, effects of Smad3 on YAP nuclear accumulation and effects of YAP silencing on Erk2 localization.

a, b) *In situ* PLA detection of the association between endogenous Smad3 and Imp7 in RPE-1 cells silenced with control, two independent siRNAs for Imp7 or one siRNA for Smad3, using anti-Imp7 (mouse) and anti-Smad3 (rabbit) antibodies; quantification of the PLA signal is shown in panel b. For each sample, N = 9 fields from 3 independent experiments. From left to right, p-value = 7.35e-07, 1.27e-05 and 5.49e-07.

c, d) *In situ* PLA detection of the association between endogenous Smad3 and Imp7 in RPE-1 cells treated with (+) or without (-) TGFβ, using anti-Imp7 (mouse) and anti-Smad3 (rabbit) antibodies; quantification of the PLA signal is shown in panel d. For each sample, N = 11 fields from 3 independent experiments. P-value = 1.70e-05.

e, f) *In situ* PLA detection of the association between endogenous YAP and Imp7 in RPE-1 cells silenced with control or Smad3 siRNA, using anti-YAP (mouse) and anti-Imp7 (rabbit) antibodies. Quantification of the PLA signal is shown (f). For each sample, N = 9 fields from 3 independent experiments. P-value = 0.311.

g, h) Immunofluorescence of endogenous YAP and nuclei in RPE-1 silenced with Smad3 siRNA treated with (+) or without (-) TGFβ. Quantification is shown in the graph (h). From left to right in the graph, N = 36, 38, 44 and 42 cells per condition, from 3 independent experiments. P-value = 0.333 (1st vs 2nd lanes), 0.211 (1st vs 3rd lanes), 0.991 (2nd vs 4th lanes), 0.729 (3rd vs 4th lanes).

i, j) Immunofluorescence of endogenous Erk2 and nuclei in RPE-1 cells silenced with two independent siRNAs of Imp7 treated with EGF (30 nM 30 min). Quantification is shown in the graph (j). From left to right, N = 159, 165 and 127 cells per condition, from 3 independent experiments. From left to right, p-value = 1.06e-13 and 5.92e-08.

k, l) Immunofluorescence of endogenous Erk2 and nuclei in RPE-1 cells silenced with two independent siRNAs of YAP treated with EGF (30 nM 30 min). Quantification is shown in the graph (l). From left to right in the graph, N = 159, 171 and 171 cells per condition, from 3 independent experiments. From left to right, p-value = 0.00018 and 9.04e-05.

m, n) *In situ* PLA detection of the association between endogenous Erk2 and Imp7 in RPE-1 cells silenced with control or two independent siRNAs of YAP. Cells were treated with EGF (30 nM 30 min) and stained with anti-Imp7 (mouse) and anti-Erk2 (rabbit) antibodies. Quantification of the PLA signal is shown (n). For each sample, N = 5 fields from 3 independent experiments. Statistical analysis with a two-tailed unpaired t test. Data represent mean ± s.e.m. From left to right, p-value = 0.032 and 0.028. Scale bar 10 μm. P-values below or equal to 0.05, 0.01 or 0.005 were considered statistically significant and were labeled with 1, 2 or 3 asterisks respectively. Raw data available in the Source Data file.
