# Supplementary material for: Mechanical control of nuclear import by Importin-7 is regulated by its dominant cargo YAP
Source: Nat Commun. 2022 Mar 4;13:1174. doi: 10.1038/s41467-022-28693-y (PMC8897400; doi:10.1038/s41467-022-28693-y)
Supplement: Supplementary file 3 — Description of Additional Supplementary Files [file 41467_2022_28693_MOESM3_ESM.pdf]

Description of Additional Supplementary Files

Supplementary Data 1. Quantitative MS hypothesis-free analysis of nuclear and cytosolic fractions of RPE-1 cells grown at low or high confluence.

List of proteins identified in the quantitative MS hypothesis-free analysis of nuclear and cytosolic fractions of RPE-1 cells grown at low (10,417 cells/cm^2) or high (218,750 cells/cm^2) confluence. The columns show: the protein ID number (Protein), the number of peptides identified (NOP), levels in cytosol at low confluence respect of an internal control (Cyt1_low and Cyt3_low), levels in cytosol at high confluence (Cyt2_high and Cyt4_high), mean cytosolic levels at low density (Mean Cyt low), mean cytosolic levels at high density (Mean Cyt high), the difference in cytosolic levels between low and high density (Δcyt), p-value of the t test performed between low and high density (Ttest), levels in nuclei at low confluence respect of an internal control (Nuc1_low and Nuc3_low), levels in nuclei at high confluence (Nuc2_high and Nuc4_high), the difference in nuclear levels between low and high density (Δnuc), mean nuclear levels at high density (Mean nuc high), mean nuclear levels at low density (Mean Nuc low), p-value of the t test performed between low and high density (Ttest2), and the difference of cytosolic change between low and high confluence subtracted to the difference of nuclear change between low and high confluence (Z-score). Statistical analysis with a two-tailed unpaired t test.
